# Verification of DNA motifs *in Arabidopsis* using CRISPR/Cas9‐mediated mutagenesis

**DOI:** 10.1111/pbi.12886

**Published:** 2018-02-20

**Authors:** Chenlong Li, Chen Chen, Huhui Chen, Suikang Wang, Xuemei Chen, Yuhai Cui

**Affiliations:** ^1^ State Key Laboratory of Biocontrol and Guangdong Key Laboratory of Plant Resources School of Life Sciences Sun Yat‐sen University Guangzhou China; ^2^ London Research and Development Center Agriculture and Agri‐Food Canada London ON Canada; ^3^ Department of Biology Western University London ON Canada; ^4^ Department of Botany and Plant Sciences Institute of Integrative Genome Biology University of California Riverside Riverside CA USA; ^5^ Howard Hughes Medical Institute University of California Riverside Riverside CA USA

**Keywords:** DNA motif, ChIP‐seq, CRISPR/Cas9, Genome editing

## Abstract

Transcription factors (TFs) and chromatin‐modifying factors (CMFs) access chromatin by recognizing specific DNA motifs in their target genes. Chromatin immunoprecipitation followed by next‐generation sequencing (ChIP‐seq) has been widely used to discover the potential DNA‐binding motifs for both TFs and CMFs. Yet, an *in vivo* method for verifying DNA motifs captured by ChIP‐seq is lacking in plants. Here, we describe the use of clustered regularly interspaced short palindromic repeat (CRISPR)/CRISPR‐associated 9 (Cas9) to verify DNA motifs in their native genomic context in Arabidopsis. Using a single‐guide RNA (sgRNA) targeting the DNA motif bound by REF6, a DNA sequence‐specific H3K27 demethylase in plants, we generated stable transgenic plants where the motif was disrupted in a REF6 target gene. We also deleted a cluster of multiple motifs from another REF6 target gene using a pair of sgRNAs, targeting upstream and downstream regions of the cluster, respectively. We demonstrated that endogenous genes with motifs disrupted and/or deleted become inaccessible to REF6. This strategy should be widely applicable for *in vivo* verification of DNA motifs identified by ChIP‐seq in plants.

## Introduction

DNA motifs are short *cis*‐regulatory elements that are recognized by transcription factors (TFs) and chromatin‐modifying factors (CMFs) for temporal and tissue‐specific gene expression. The ChIP‐seq has been a powerful method in *de novo* discovery of potential DNA motifs bound by TFs and CMFs. However, it often generates more potential DNA motifs with a high false‐positive rate. Current methods for verifying the binding motifs identified by ChIP‐seq typically include yeast one‐hybrid (Y1H) and electrophoresis mobility shift assay (EMSA). However, both Y1H and EMSA are *in vitro* methods, and thus, results from them may not reflect DNA–protein interactions *in vivo*. Alternatively, a transgene that contains the DNA‐binding motif or variations of the motif can be transferred into the organism, and the binding ability of the TFs and/or CMFs to the motif can be evaluated by ChIP‐qPCR. However, one concern is that the transgene may not behave as endogenous target loci because the chromatin context of the insertion site of the transgene may differ from that of endogenous target loci. Therefore, an *in vivo* method for verifying DNA motifs captured by ChIP‐seq in their native chromatin context is in demand in plants.

The clustered regularly interspaced short palindromic repeat (CRISPR)/CRISPR‐associated 9 (Cas9) system has been successfully applied to efficiently edit genomes in bacteria, animals and plants (Doudna and Charpentier, 2014; Sander and Joung, 2014). The CRISPR loci are variable short spacers separated by short repeats, which are transcribed into synthetic single‐guide RNA (sgRNA) that forms a functional complex with the Cas9 nuclease (Mali *et al*., 2013). The sgRNA guides the Cas9 to genomic loci matching a 20‐bp complementary DNA, making a DNA double‐strand break (DSB) immediately upstream of a required protospacer adjacent motif (PAM) (Ma *et al*., 2016). The DSB can be repaired by nonhomologous end‐joining pathway (NHEJ), which is error prone, creating insertions and/or deletions (Symington and Gautier, 2011). Recent studies have successfully applied the CRISPR/Cas9 system to verify DNA motifs in their native genomic context in mouse and human (Kim and Kim, 2017; Tanimura *et al*., 2016); however, such a strategy has yet to be tested in plants.

In this study, we set out to use the CRISPR/Cas9 system to verify DNA motif in plants. We and others previously reported that the plant H3K27 demethylase RELATIVE OF EARLY FLOWERING 6 (REF6), unlike most CMFs, has an intrinsic DNA‐binding ability (Cui *et al*., 2016; Li *et al*., 2016). Genomewide binding analysis by ChIP‐seq showed that the CTCTGYTY (Y represents C or T) DNA motif, either single or multiple copies, is enriched at REF6‐binding sites supporting the notion that the CTCTGYTY motif is crucial for recruiting REF6 to its target loci. As a proof of principle, we used the CRISPR/Cas9 system to target the CTCTGYTY DNA motif in *Arabidopsis*. We found that motif deletions at two different REF6 target genes were accompanied by the loss of bindings of REF6 *in vivo*, demonstrating that CRISPR can be used for functional verification of DNA motifs identified by ChIP‐seq in plants.

## Results and discussion

**Figure 1:**
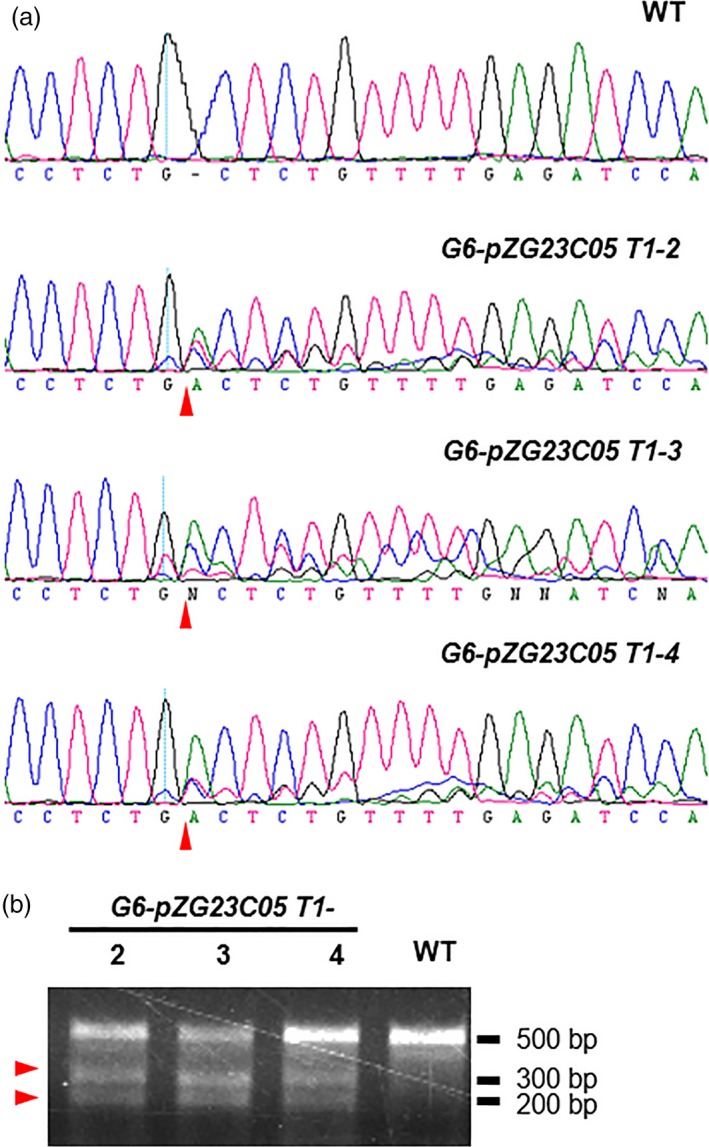
Targeted editing of the CTCTGYTY motif from an endogenous REF6 target gene by CRISPR/Cas9 system. (a) DNA sequencing peaks showing the successful gene editing in the target region of *AT5G61250* in three representative T1 lines. The sequencing result from WT is served as the negative control. Red triangles point to the putative cutting sites by Cas9. (b) T7EI assay showing the successful gene editing in the target region of *AT5G61250*. Red triangles point to the two bands at expected size after T7EI digestion.

To remove the CTCTGYTY motif at its endogenous sites using CRISPR/Cas9 for functional verification, we took two different strategies, targeting single‐ and multiple‐motif‐containing loci, respectively. For REF6 target loci that contain a single CTCTGYTY motif, we used sgRNAs that meet the following two additional criteria to increase the success rate of disruption: (i) the recognition site of the sgRNA covers the motif and (ii) the predicted cutting site, which is between third and fourth nucleotides upstream of the PAM sequence, is located in the motif. We selected four single‐motif‐containing REF6 target genes and designed a sgRNA for each of them (Figure S1 and Table S1). The AGI numbers of the four REF6 target genes are *AT* *5G* *61250*,* AT4* *G1* *6400*,* AT4* *G*3 *0620* and *AT5* *G5* *2170*, and the four gRNAs were named as G6, G1, G3 and G5, respectively. We individually integrated the four sgRNAs (driven by the *AtU6* promoter) into the *pZG23C05* vector carrying Basta (driven by the *35S* promoter) and *Cas9* (driven by the *Ubi* promoter) expression cassettes (Figure S2a). The four resulting vectors were individually transformed into the *pREF6::REF6‐GFP ref6‐1* transgenic *Arabidopsis* plants that express GFP fused with REF6 under the control of its native promoter (Li *et al*., 2016). To examine the gene‐editing efficacy of the four sgRNAs, sequences containing the targeting sites of sgRNAs were amplified by PCR using genomic DNA from the T1 plants. We sequenced PCR products from 37, 57, 45 and 39 T1 plants transformed by G6, G1, G3 and G5 sgRNAs, respectively. Gene‐editing events were observed in 34 of 37 T1 plants transformed by G6 sgRNA, but not in those transformed by the other three sgRNAs (Figure 1a and Table S1). We then confirmed editing by PCR sequencing and T7EI experiments in three selected G6‐transformed plants (T1‐2, T1‐3 and T1‐4) (Figure 1).

**Figure 2:**
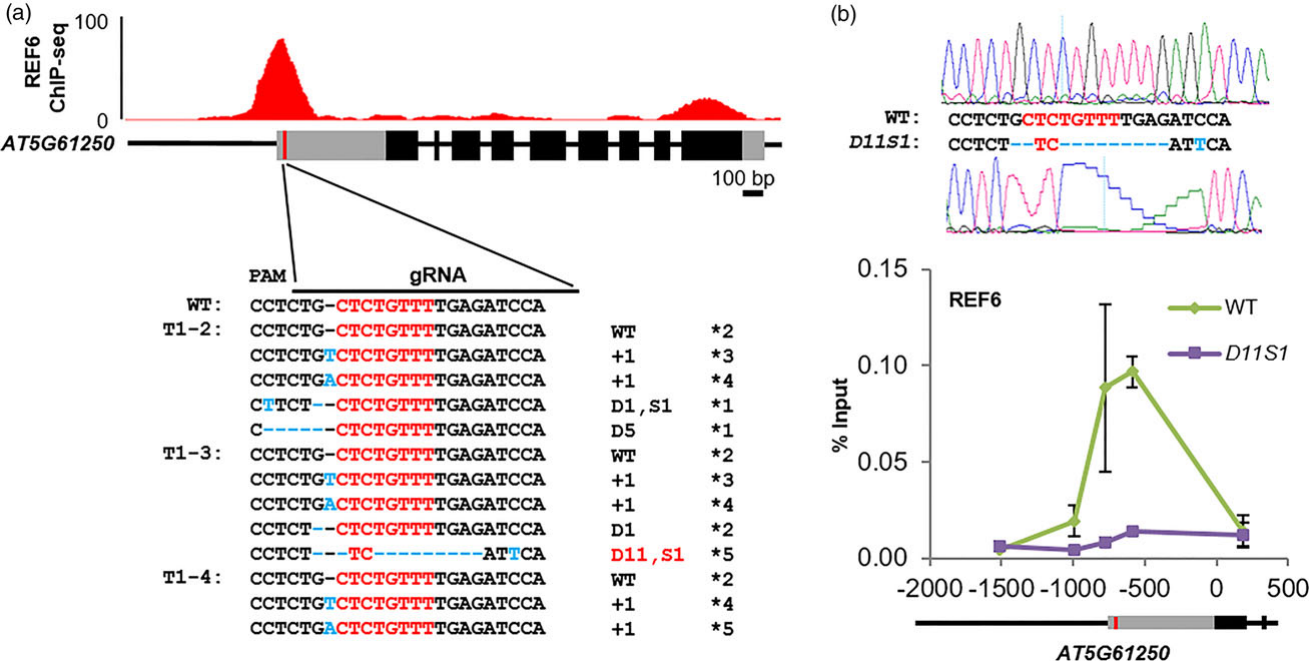
CRISPR/Cas9‐mediated disruption of the CTCTGYTY motif for functionally verifying the DNA‐binding motif *in vivo*. (a) Sequencing results of alleles of *AT5G61250* from three representative T1 transgenic plants (T1‐2, T1‐3 and T1‐4). On the top, ChIP‐seq genome‐browser view of REF6 binding at *AT5G61250* locus. Schematic representation of *AT5G61250* genomic locus is shown underneath. Black and grey boxes represent exons and UTRs, respectively. The red vertical line indicates the position of the CTCTGYTY motif in *AT5G61250* locus. The CTCTGYTY motif is shown in red font. On the right, plus (+) signs, letter D and letter S indicate the number of nucleotides inserted, deleted and replaced, respectively. The asterisks indicate the numbers of independent clones sequenced. (b) ChIP‐qPCR results showing the binding of REF6 at WT and *D11S1* alleles of *AT5G61250* locus. ChIP signals are shown as percentage of input. Error bars indicate standard deviations among three biological replicates. Schematic representation of part of the *AT5G61250* genomic locus is shown underneath. Black and grey boxes represent exons and UTRs, respectively. The red vertical line labelled the position of the CTCTGYTY motif. On the top, sequencing results for WT and *D11S1* alleles.

To check whether the editing events in the G6 transgenic plants disrupted the CTCTGYTY motif, we cloned and sequenced the PCR fragments from three representative T1 transgenic plants (T1‐2, T1‐3 and T1‐4). Several different types of mutations were detected in these three lines (Figure 2a). The mutations found in lines T1‐2 and T1‐4 did not disrupt the CTCTGYTY motif (Figure 2a). However, we found a clone from line T1‐3 that contained an 11 nucleotide deletions and an A to T substitution (Figure 2a), causing the disruption of the CTCTGYTY motif (CTCTGTTT to TC). We named this mutant allele as *D11S1* (D represents Deletion and S represents Substitution). The T1‐3 plant was selfed, and the T2 progenies were analysed by PCR sequencing. Among the nineteen tested T2 plants, a homozygous *D11S1* mutant plant was found (Figure 2b). Together, our results indicate that targeted disruption of a DNA motif can be achieved by careful design of sgRNAs.

We then assessed the occupancy of REF6 at the *D11S1* mutant locus in the T3 generation. By ChIP‐qPCR, we found that, compared to the strong binding of REF6 around the CTCTGYTY motif at the wild‐type allele of *AT5G61250*, the binding of REF6 was completely diminished at the *D11S1* mutant locus where the CTCTGYTY motif in *AT5G61250* was disrupted (Figure 2b), demonstrating that the motif is necessary for the binding of REF6. To investigate whether off‐target mutations might have happened in the *D11S1* line, we examined the four predicted most likely off‐target sites. Off‐target mutations were not detected at any of the four sites (Table S2). Therefore, the G6 sgRNA used in this experiment is specific to the REF6 target gene *AT5G61250*.

**Figure 3:**
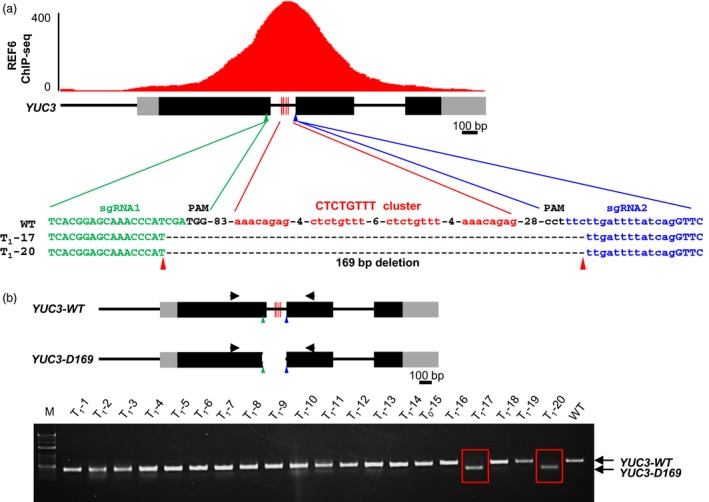
Targeted deletion of the cluster of the four CTCTGYTY motifs at *YUC3* by the *YAO* promoter‐based CRISPR/Cas9 system. (a) Sequencing results showing the deletion mutant alleles of *YUC3* from two T1 transgenic plants (T1‐17 and T1‐20). On the top, ChIP‐seq genome‐browser view of REF6 binding at *YUC3* locus. Schematic representation of *YUC3* genomic locus is shown underneath. Black and grey boxes represent exons and UTRs, respectively. The four red vertical lines indicate the position of the cluster of the CTCTGYTY motifs in *YUC3* locus. The four CTCTGYTY motifs are shown in red font. (b) PCR amplification results showing deletion of the 169‐bp DNA from *YUC3* in two of the twenty T1 lines. Schematic representations of the WT and *D169* alleles of *YUC3* are shown on the top. Black and grey boxes represent exons and UTRs, respectively. The four red vertical lines mark the position of the CTCTGYTY motifs in *YUC3* locus. The green and blue triangles indicate the putative cutting sites of the two sgRNAs. The black triangles indicate the positions of PCR primers. WT is served as the negative control. Red boxes mark the two lines with genomic fragment deleted. M, DNA size marker ladder.

Yet, many REF6‐binding sites contain multiple CTCTGYTY motifs that form a cluster (Cui *et al*., 2016; Li *et al*., 2016). To demonstrate that CRISPR/Cas9 is robust for DNA motif verification, we decided to delete the whole cluster of the CTCTGYTY motifs from *YUC3*, an REF6 target locus that has four repeats of the motif (Li *et al*., 2016). We designed two sgRNAs that target the sequences upstream and downstream of the cluster, respectively (Figure 3a). Deleting large genomic DNA fragments by two sgRNAs requires that the two cutting reactions occur simultaneously in the same cell. Previous studies have suggested that expressing *Cas9* in meristems and embryonic cells at high levels is key to achieving high efficiency in gene editing in *Arabidopsis* (Hyun *et al*., 2015; Wang *et al*., 2015; Yan *et al*., 2015, 2016). To increase the efficiency of the deletion mediated by two sgRNAs, we used the *YAO* promoter‐based CRISPR/Cas9 system, which was reported to have much higher efficiency on generating heritable mutations (Yan *et al*., 2015). The *YAO* gene promoter is highly expressed in the embryo sac, embryo, endosperm and pollen (Li *et al*., 2010). The two sgRNAs were cloned into the *pYAO: hSpCas9* vector (Figure S2b), and the construct was then introduced into wild‐type (WT) plants. The T1 transgenic lines were analysed by PCR using a pair of primers that amplifies the genomic DNA spanning the targeted motif cluster. From 20 T1 transgenic plants, we identified two plants (T1‐17 and T1‐20) that showed only the expected smaller PCR band (Figure 3b). Sequencing of the PCR products revealed that there was a deletion of 169‐bp DNA encompassing the whole cluster of four CTCTGYTY motifs (Figure 3a). This *YUC3* mutant allele was named as *YUC3‐D169*.

**Figure 4:**
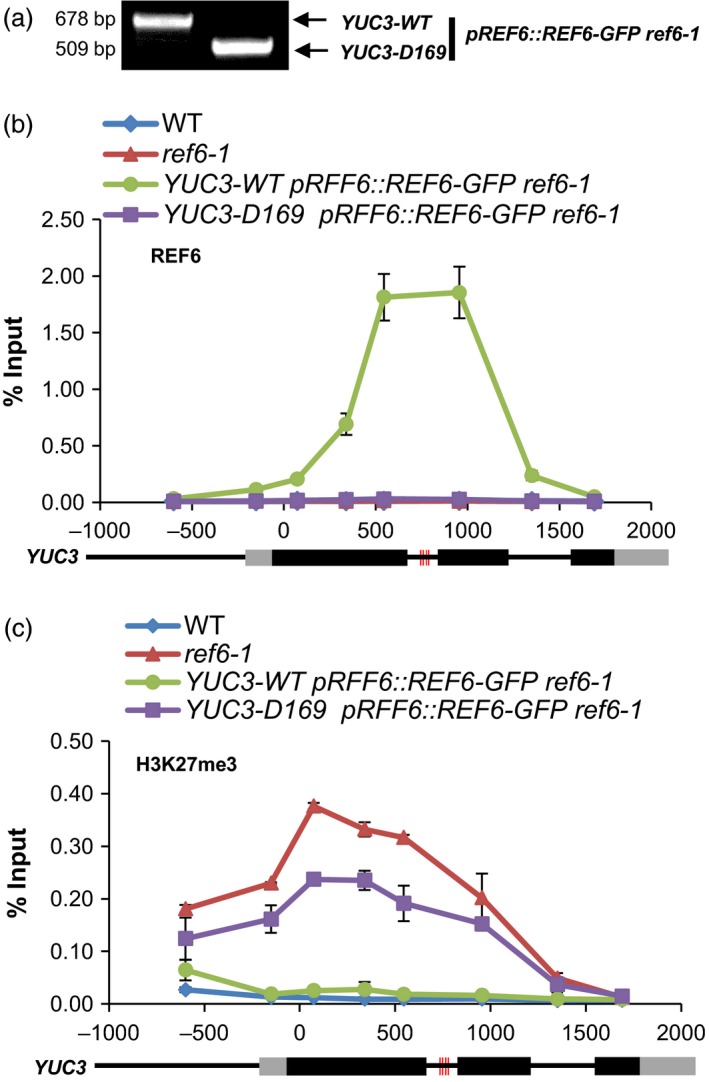
CRISPR/Cas9‐mediated deletion of the cluster of CTCTGYTY motifs to functionally verify the DNA‐binding motif *in vivo*. (a) PCR amplification results showing the homozygous *D169* allele of *YUC3* were introduced into *pREF6::REF6‐GFP ref6‐1*. (b) ChIP‐qPCR results showing the binding of REF6 at WT and *D169* alleles of *YUC3* locus. ChIP signals are shown as percentage of input. Error bars indicate standard deviations among three biological replicates. Schematic representation of *YUC3* genomic locus is shown underneath. Black and grey boxes represent exons and UTRs, respectively. (c) ChIP‐qPCR results showing the level of H3K27me3 at WT and *D169* alleles of *YUC3* locus. ChIP signals are shown as percentage of input. Error bars indicate standard deviations among three biological replicates. Schematic representation of the *YUC3* genomic locus is shown underneath. Black and grey boxes represent exons and UTRs, respectively. The red vertical lines indicate the CTCTGYTY motifs in *YUC3* locus.

To assess the effect of the deletion of the cluster on the binding of REF6 at *YUC3*, the *YUC3‐D169* mutant allele was introduced into the *pREF6::REF6‐GFP ref6‐1* plants by genetic crossing (Figure 4a). Examination of the most likely predicted off‐target sites of each of the sgRNAs by PCR sequencing did not detect off‐target mutations, suggesting that both sgRNAs are specific to the *YUC3* locus only (Table S2). By ChIP‐qPCR, we found that REF6 was unable to bind to *YUC3‐D169*, while it was strongly enriched at the cluster of motifs in wild‐type *YUC3* (Figure 4b). Furthermore, we wondered whether the H3K27 demethylase activity of REF6 at its target genes requires the CTCTGYTY motif. As shown in Figure 4c, loss of REF6 led to the ectopic accumulation of H3K27me3 at *YUC3*, which was eliminated upon the introduction of the *pREF6::REF6‐GFP* transgene. However, the elimination of H3K27me3 by *pREF6::REF6‐GFP* was not found at *YUC3‐D169*, suggesting that deletion of the CTCTGYTY motifs prevents the recruitment of REF6 and consequently its H3K27me3 demethylation activity at this target locus (Figure 4c). Taken together, our CRISRP/Cas9‐mediated disruption (for *AT5G61250*) as well as deletion (for *YUC3*) of the motifs followed by ChIP‐qPCR analysis validated the CTCTGYTY sequence as the binding motif of REF6. Consistently, REF6‐mediated H3K27me3 demethylation at REF6 target genes is dependent on the CTCTGYTY motif.

## Conclusions

In this work, we have demonstrated that CRISPR/Cas9‐mediated strategies can be employed to functionally verify the DNA motifs captured by ChIP‐seq in its native genomic context in plants. As a proof‐of‐principle, we have successfully used such strategies to disrupt or delete the CTCTGYTY motifs from REF6 target genes found by ChIP‐seq and observed the loss of occupancy of REF6 *in vivo*. Although we only tested the REF6‐binding motif, other *cis* DNA motifs recognized by TF and/or CMFs should also be able to be verified using this approach. During the course of preparing this article, a report demonstrated the use of CRISPR/Cas9 system to modify promoters of target genes in tomato to produce numerous consecutive variations for enhancing breeding (Rodriguez‐Leal *et al*., 2017). Among the five tested REF6 target genes, two of them were successfully edited by CRISPR/Cas9 and the binding by REF6 was verified, suggesting the overall efficiency of the editing mediated by sgRNAs is relatively high. However, a quick testing of the efficiency of candidate sgRNAs can be performed in protoplast before generating stable transgenic plants. As ChIP‐seq experiments typically discover thousands of target loci, it should not be difficult to find suitable sgRNAs (i.e. PAM sequence requirement) for targeting the motifs for disruption/deletion. In conclusion, we expect that CRISPR should be widely applicable for *in vivo* verification of the potential DNA motifs discovered by ChIP‐seq in plants.

## Materials and methods

### Plant materials and growth conditions

*Arabidopsis* seeds were stratified for 4 days at 4 °C in darkness. Then, the seeds were sown on soil or on agar plates containing 4.3 g/L Murashige and Skoog nutrient mix (Sigma‐Aldrich), 1.5% sucrose (pH 5.8) and 0.8% agar. Plants were grown in growth rooms with 16‐h light/8‐h dark cycles at 22 °C. The *ref6‐1* (SALK_001018) mutants and the *ProREF6:REF6‐GFP ref6‐1* transgenic plants have been described previously (Li *et al*., 2016).

### Construction of Cas9 and sgRNA expression vectors

For the four single‐motif‐containing REF6 target genes, pairs of oligonucleotides (Table S3) including the targeting sequences were synthesized as primers, annealed and cloned into *pZG23C05* vector according to the manufacturer's protocol (ZGene Biotech Inc.).

For deleting the CTCTGYTY cluster in *YUC3*, the *Yao* promoter‐based CRISPR/Cas9 system (Yan *et al*., 2015) was used. In brief, a pair of sgRNAs (Table S3) targeting sequence upstream and downstream of the CTCTGYTY cluster in *YUC3* was designed. The first sgRNA was cloned into *BsaI*‐digested *AtU6‐26‐sgRNA‐SK* vector. The resulting vector was then double‐digested with *SpeI* and *NheI*, separated on agarose gel, and the lower DNA fragment was purified. The second sgRNA (Table S3) was also cloned into the *AtU6‐26‐sgRNA‐SK* vector and then linearized by *SpeI*. The purified lower DNA fragment containing the first sgRNA was cloned into the linearized vector so that the two sgRNAs were cloned into one vector. Then, this vector was double‐digested again by *SpeI* and *NheI*. The smaller DNA fragment containing the two sgRNAs was purified and cloned into *SpeI*‐digested *pCAMBIA1300‐pYAO: Cas9* to generate the final transgene construct for *Arabidopsis* transformation.

### Generation of transgenic plants

The constructs were introduced into *Agrobacterium tumefaciens* GV3101, which was then used to transform *ref6‐1 ProREF6:REF6‐GFP* or WT plants using the floral dip method (Clough and Bent, 1998). The transgenic seeds from the T1 generation were screened on MS plates with 50 μg/L of glufosinate or 25 μg/L hygromycin.

### Genotyping

Genomic DNA was extracted from leaves of the transgenic plants and used for PCR to amplify the genomic fragments containing the sgRNA targeting sites. The PCR products were either directly sequenced or cloned into pGEM^®^‐T Easy vector (Promega). Bacterial colony PCR was conducted, and positive clones were picked for sequencing. Primer sequences used are listed in Table S3.

For the T7EI assay, 8 μL of PCR products was mixed with 2 μL of 10 × NEB buffer 2 and annealed using the following condition: 95 °C for 5 min, ramp down to 85 °C at −2 °C/s, ramp down to 20 °C at −0.2 °C/s and 4 °C for 5 min. Then, 0.5 μL of T7EI (NEB) was added and incubated at 37 °C for 30 min. The reactions were loaded on 2% agarose gel.

### ChIP assay

ChIP was carried out as described (Gendrel *et al*., 2005; Li *et al*., 2015) with minor modifications. Briefly, two grams of 14‐day‐old seedlings grown on MS agar was harvested and cross‐linked with 1% formaldehyde for 20 min under vacuum and then ground into fine powder in liquid nitrogen. Chromatin was isolated and sheared into 200‐ to 800‐bp fragments by sonication. The sonicated chromatin was incubated with 5 μL of anti‐GFP (Abcam, ab290) or anti‐H3K27me3 (Millipore, 07‐449) antibodies overnight at 4 °C. The precipitated DNA was then recovered with the MinElute PCR Purification Kit (Qiagen) according to the manufacturer's instructions. ChIP‐qPCR was performed with three technical replicates, and results were calculated as percentage of input DNA according to the Champion ChIP‐qPCR user manual (SABioscience). ChIP experiments were performed at least three times. Primer sequences used for ChIP‐qPCR are listed in Table S3.

### ChIP‐seq data analyses

The ChIP‐seq data for genomewide binding of REF6 were previously described (Li *et al*., 2016) and have been deposited in Gene Expression Omnibus (GEO) under the accession code GSE72736. The binding peaks of REF6 were first converted to Wiggle (WIG) files using MACS (Zhang *et al*., 2008), which were imported to Integrated Genome Browser (IGB) (Nicol *et al*., 2009) for visualization.

## Authors’ contributions

C.L. conceived the project. C.C., H.C., S.W. and C.L. performed experiments. C.C. and C.L. conducted bioinformatics analyses. C.L. analysed data. C.L., X.C. and Y.C. wrote the manuscript.

## Competing financial interests

The authors declare no competing financial interests.

## Supporting information


**Figure S1** Schematic representation of the four sgRNAs and the corresponding single‐motif‐containing REF6 target genes.

**Figure S2** Schematic representation of CRISPR/Cas9 vectors used in this study.

**Table S1** Summary of the sgRNAs used for single‐motif‐containing REF6 target loci.

**Table S2** The potential off‐target sites of the gRNAs.

**Table S3** Primers used in this study.
