# Supplementary figures and images for: Verification of DNA motifs in Arabidopsis using CRISPR/Cas9‐mediated mutagenesis
Source: Plant Biotechnol J. 2018 Feb 20;16(8):1446–51. doi: 10.1111/pbi.12886 (PMC6041440; doi:10.1111/pbi.12886)

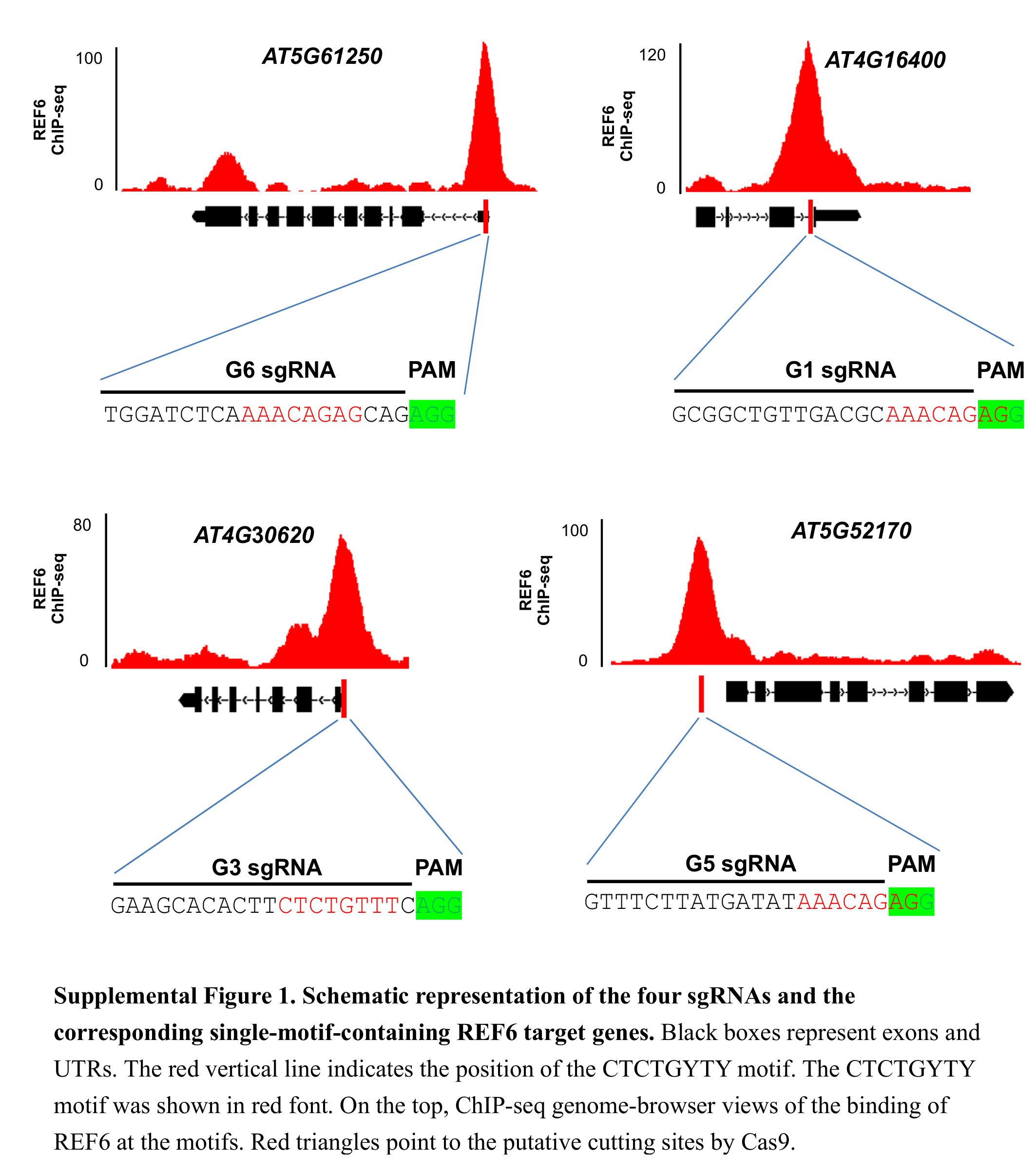

Supplement: Supplementary file 1 — Figure S1 Schematic representation of the four sgRNAs and the corresponding single‐motif‐containing REF6 target genes. [file PBI-16-1446-s004.tiff]

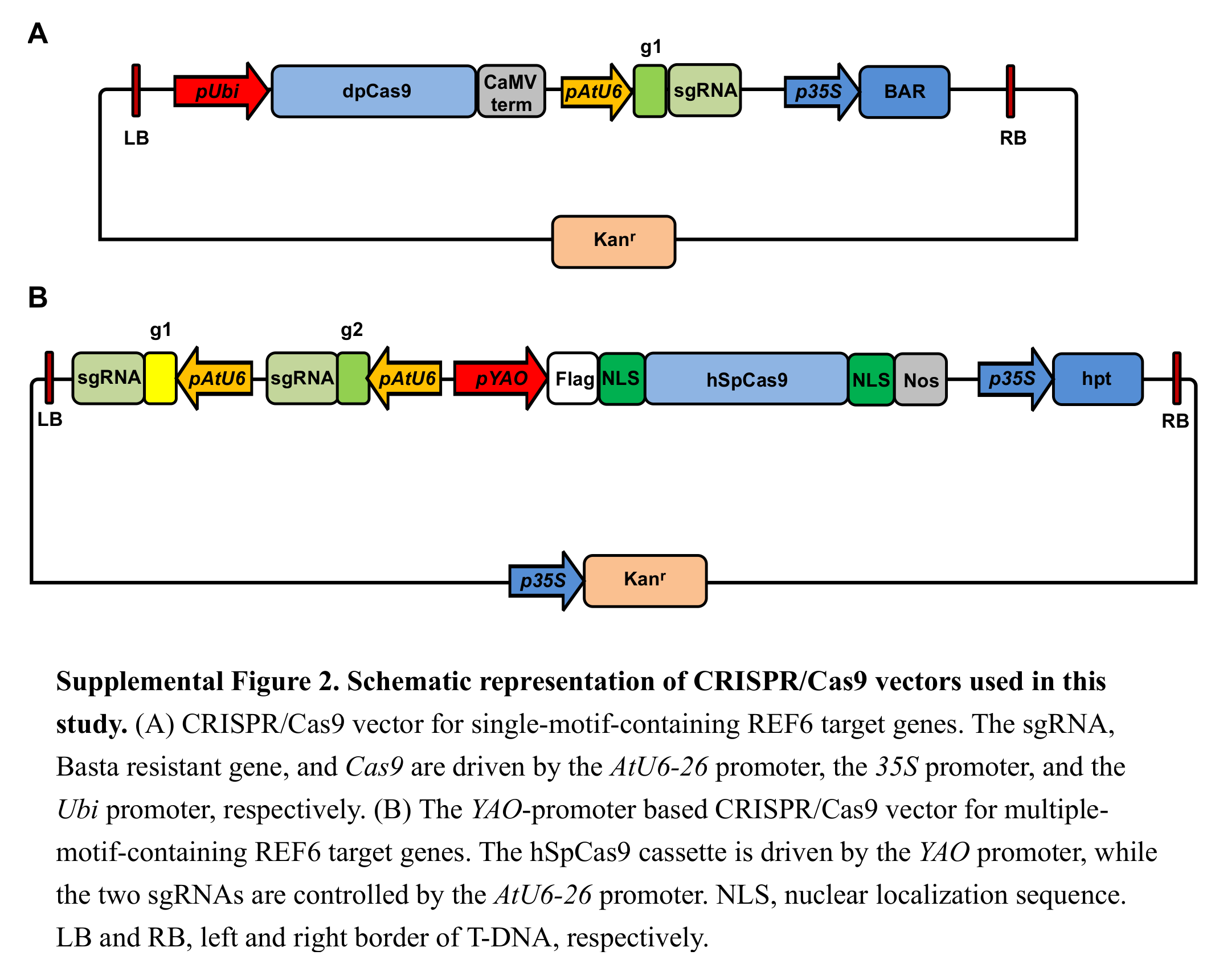

Supplement: Supplementary file 2 — Figure S2 Schematic representation of CRISPR/Cas9 vectors used in this study. [file PBI-16-1446-s003.tiff]

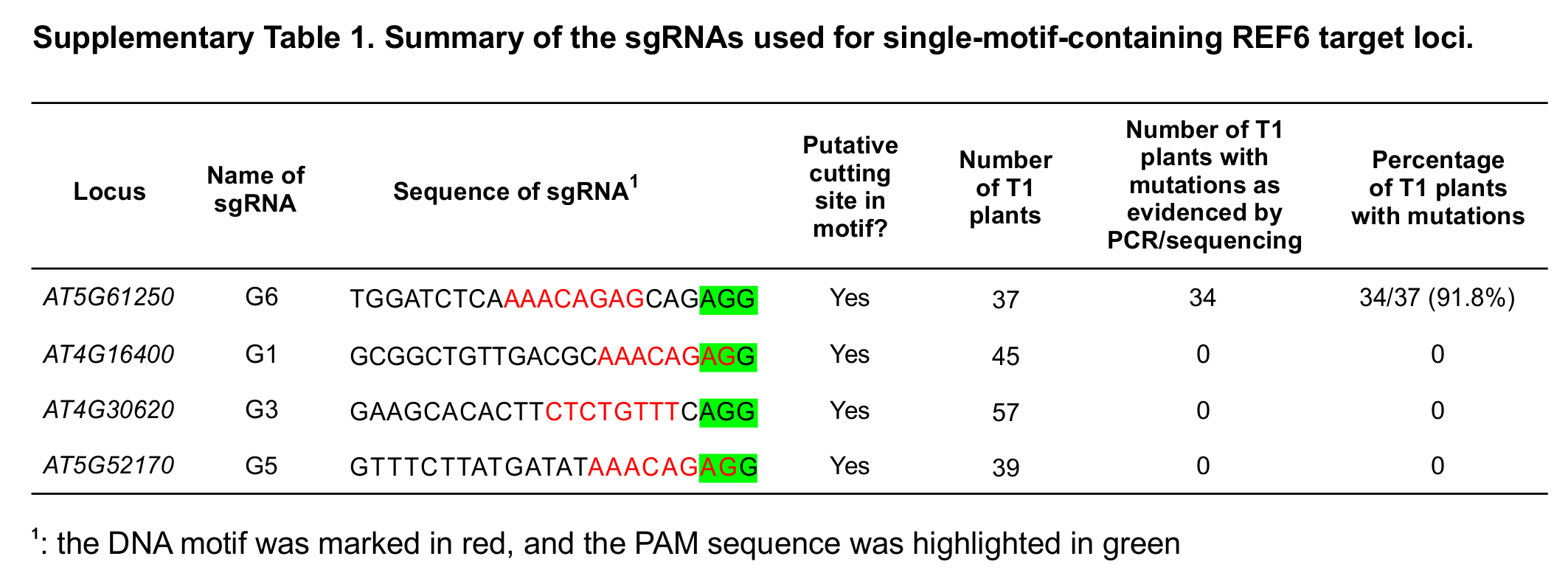

Supplement: Supplementary file 3 — Table S1 Summary of the sgRNAs used for single‐motif‐containing REF6 target loci. [file PBI-16-1446-s002.tiff]

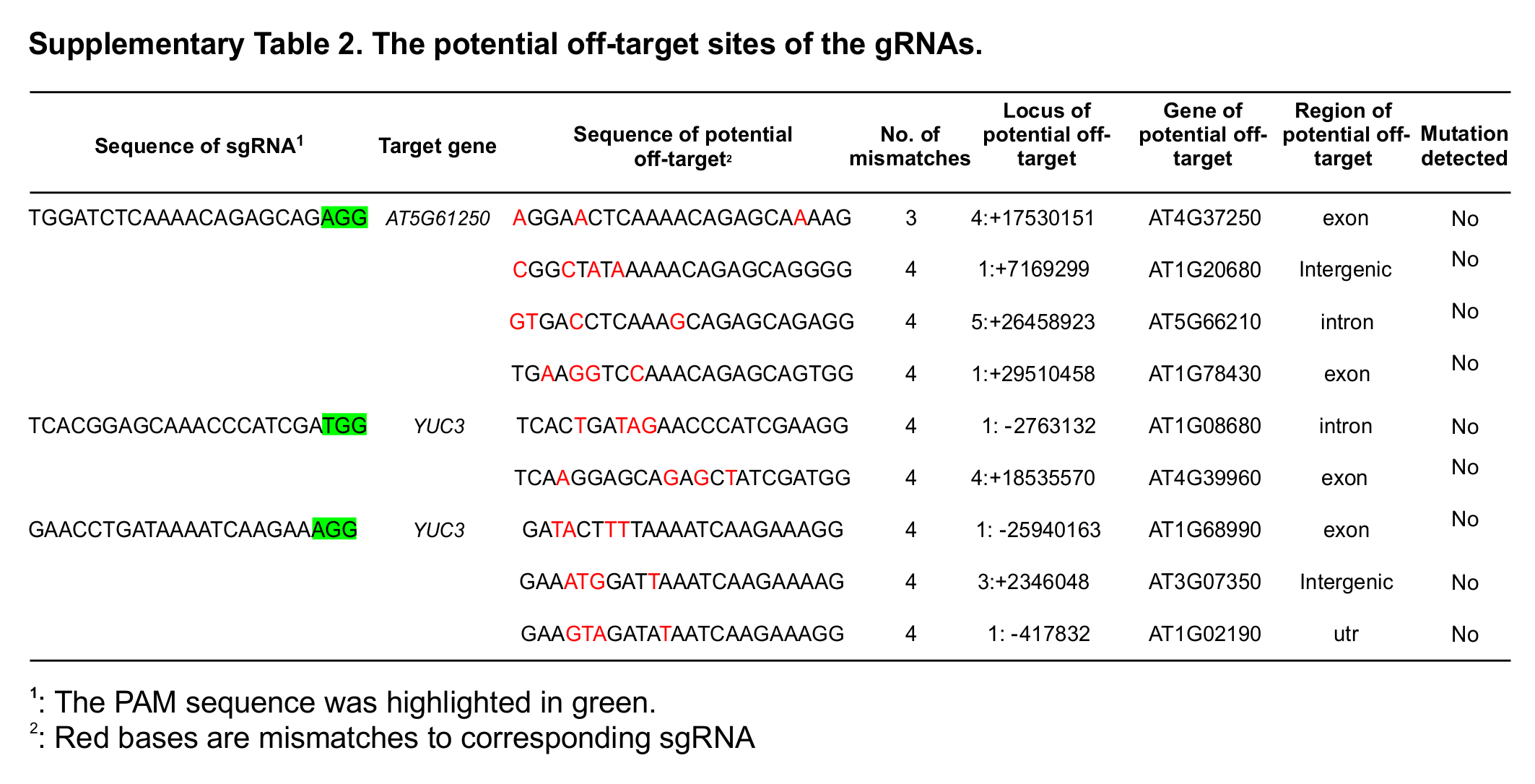

Supplement: Supplementary file 4 — Table S2 The potential off‐target sites of the gRNAs. [file PBI-16-1446-s005.tiff]
