# Supplementary material for: Verification of DNA motifs in Arabidopsis using CRISPR/Cas9‐mediated mutagenesis
Source: Plant Biotechnol J. 2018 Feb 20;16(8):1446–51. doi: 10.1111/pbi.12886 (PMC6041440; doi:10.1111/pbi.12886)
Supplement: Supplementary file 5 — Table S3 Primers used in this study. [file PBI-16-1446-s001.docx]

**Primers for generating sgRNAs:**

AT5G61250-target-F: TTGTGGATCTCAAAACAGAGCAG

AT5G61250-target-R: AACCTGCTCTGTTTTGAGATCCA

AT4G16400-target-F: TTGGCGGCTGTTGACGCAAACAG

AT4G16400-target-R: AACCTGTTTGCGTCAACAGCCGC

AT4G30620-target-F: TTGGAAGCACACTTCTCTGTTTC

AT4G30620-target-R: AACGAAACAGAGAAGTGTGCTTC

AT5G52170-target-F: TTGGTTTCTTATGATATAAACAG

AT5G52170-target-R: AACCTGTTTATATCATAAGAAAC

YUC3-Yao-F1: ATTGTCACGGAGCAAACCCATCGA

YUC3-Yao-R1: AAACTCGATGGGTTTGCTCCGTGA

YUC3-Yao-F2: ATTGAACCTGATAAAATCAAGAA

YUC3-Yao-R2: AAACTTCTTGATTTTATCAGGTT

**Primers for amplifying genomic regions containing sgRNA targeting sites:**

AT5G61250-crispr-F: TGGGCTCCTTAGATTAGAAACTG

AT5G61250-crispr-R: TGGTGTCAGAGAAGCATGGA

AT4g16400-crispr-F: GGACTTGAGAACTTGCGTTGA

AT4g16400-crispr-R: TGTTACACAATCATTCGACATTTT

AT4G30620-crispr-F: AGTGAAACAGGAGAAGAAGCAAC

AT 4G30620-crispr-R: GCTCGCATGGTAATCACACT

AT5G52170-crispr-F: GTGATGGATCAAGAAACGGCTC

AT5G52170-crispr-R: GCTCAGACAAGAAGCAACCTC

YUC3-crispr-678F: CTGTGGCGGGTTAAGACCAT

YUC3-crispr-678R: GGGTAACGCTCCGATGTCAA

**Primers for amplifying genomic regions of off-target sites:**

For At5G61250 gRNA off-target 1: AT4G37250

AT4G37250-off-F: ACGAAGCTCTCTAGCGTTGAA

AT4G37250-off-R: GCAGGAAGTGGAGTGTTTGGT

For At5G61250 gRNA off-target 2: AT1G20680

AT1G20680-off-F:ACGTCACTTTTGTGGTTTGACA

AT1G20680-off-R:AGATGCCCCACTTATGACCCT

For At5G61250 gRNA off-target 3: AT5G66210

AT5G66210-off-F: TCCTATCCAGCAATTCGCCTC

AT5G66210-off-R: GCTACACCTACGTCGCCATC

For At5G61250 gRNA off-target 4: AT1G78430

AT1G78430-off-F: AGCGAGGAGAATGAGACCTTG

AT1G78430-off-R: TCACTTCATTTCTGCCCTTTCTT

For YUC3 gRNA1 off-target 1: AT1G08680

AT1G08680-off-F: ACCAGCGTACGATACTGACA

AT1G08680-off-R: TGATGGGTTTCAGCTTCTGTGA

For YUC3 gRNA1 off-target 2: AT4G39960

AT4G39960-off-F:GACAGATATGGCGAGGCAGG

AT4G39960-off-R:TGTTGGAATACACCGAGTGGT

For YUC3 gRNA2 off-target 1: AT1G68990

AT1G68990-off-F: TGCAGCTATACCGCCAAAGAG

AT1G68990-off-R: CCCGATCTGAGTAGCAGCATT

For YUC3 gRNA2 off-target 2: upstream of AT3G07350

AT3G07350-off-F: GTTGTTCTCTCACTCTCCCACG

AT3G07350-off-R: ATGATTTATGGTGGGGGCCA

For YUC3 gRNA2 off-target 3: AT1G02190

AT1G02190-off-F: CATGCGGGGTAAGGACCAT

AT1G02190-off-R: CATGCACTTGAGGGTTGGGA

**Primers for ChIP-qPCR:**

599--514-YUC3-ChIP-F: TTATCTGGTTTCGACTTGGACT

599--514-YUC3- ChIP-R: ATGGTCGGTTCAGACCGAAT

151--17-YUC3- ChIP-F: CCAATCCAACAGAACACACACAT

151--17-YUC3-ChIP-R: AGTTTGCTTGTCTGGTTGTGAG

73-151-YUC3-ChIP-F: GACATTTTCTCCCGGCGTTG

73-151-YUC3-ChIP-R: CAGCAACGGCTAGACCTGAT

340-470-YUC3-ChIP-F: CTTGAGTCCTACGCAGCCAA

340-470-YUC3-ChIP-R: CAAGAACCGAGCTGACCCAT

549-649-YUC3-ChIP-F: TCTAGAGGATTTTGGCGGCG

549-649-YUC3-ChIP-R: CGGAGTTTCCACATCCCACC

AT1G04610-ChIP-F: TGGCTCGCGGACAAGACTA

AT1G04610-ChIP-R: TTTATGATTCCGGGGACGATTTT

1350-1514-YUC3-ChIP-F: CCCGACCCGGTCCTAGTTTA

1350-1514-YUC3-ChIP-R: AGTCCTAACTGTCATTACCGGA

1690-1881-YUC3-ChIP-F: GTGCGTCGCTTGATGCTATG

1690-1881-YUC3-ChIP-R: TTCTTCCGAGGAGAGGGCTT

1516--1389-5G61250-ChIP-F: TGCATGAATAATCGGTCACGA

1516--1389-5G61250-ChIP-R: GCCAAAATATCCTCCGAGCG

992--826-5G61250-ChIP-F: TTGAGAAGACAGAGCCCACA

992--826-5G61250-ChIP-R: GCAAATGTTACAAAGGAGCCCA

775--697-5G61250-ChIP-F: TTCGGTTTGGTGTAAACGGT

775--697-5G61250-ChIP-R: TGGAGTTGACAACACTTCATCA

AT5G61250-ChIP-Q-F: TCTGAGGTCAGTTTCTGTTCTTGA

AT5G61250-ChIP-Q-R: AGGGCATTAGTTGACTTTTTGGA

184-334-5G61250-ChIP-F: CAATGTCCTTGGGGTTACGC

184-334-5G61250-ChIP-R: AGAGGAGAAGCTAAGTTCTGAGT
